# Division of Responsibility in Child Feeding and Eating Competence among Brazilian Caregivers

**DOI:** 10.3390/nu15092225

**Published:** 2023-05-08

**Authors:** Rafaella Dusi, Raquel Braz Assunção Botelho, Eduardo Yoshio Nakano, Fabiana Lopes Nalon de Queiroz, Renata Puppin Zandonadi

**Affiliations:** 1 University of Brasília, Faculty of Health Sciences, Department of Nutrition, Campus Universitario Darcy Ribeiro, Brasilia 70910-900, Brazil; 2 University of Brasília, Department of Statistics, Campus Universitario Darcy Ribeiro, Brasilia 70910-900, Brazil

**Keywords:** infant feeding, division of responsibility in feeding, eating competence, Brazilian children, caregivers

## Abstract

This cross-sectional study aimed to assess Brazilian child caregivers’ eating competence (EC) and their adherence to the division of responsibility (sDOR) in child feeding. The research had national coverage in all Brazilian regions. The sample comprised 549 caregivers of Brazilian children (24 up to 72 months) recruited by social media (snowball method). Data on sDOR and EC were collected using the sDOR.2-6y^TM^ Portuguese—Brazil (sDOR.2-6y-BR) and ecSI2.0^TM^BR, both instruments validated to the Brazilian population. The scores of the sDOR.2-6y-BR were described in terms of means, standard deviations (SD), medians, and interquartile range. Student’s *t*-test and analysis of variance (ANOVA) followed by Tukey’s post hoc tests were used to compare the scores of sDOR.2-6y-BR and ecSI2.0^TM^BR with interest variables. The association between the sDOR.2-6y-BR and ecSI2.0^TM^BR scores was verified by Pearson’s correlation coefficient. Most of the participants were female (*n* = 88.7%), 37.8 ± 5.1 y/o, had a high schooling level (70.31%), and high monthly income (more than 15 minimum wages—MW) (31.69%). The children for whom the participants were responsible were mostly girls (53.19%), with an average age of 3.6 ± 1.3 y/o. The instrument presented good responsiveness (floor and ceiling effects = 0%). Cronbach’s Alpha = 0.268. There was no statistical difference in sDOR.2-6y-BR scores between caregiver’s gender, age, level of education, number of people living in the household, or by gender or age of the child. Caregivers who reported (*n* = 100) that their children had some medical diagnosis (e.g., food allergy, autism, or Down syndrome) had lower sDOR adherence scores than caregivers who indicated their children had no medical diagnosis (*p* = 0.031). There was no statistical difference in ecSI2.0^TM^BR scores between the categories of caregiver’s gender, age, occupancy, and child’s gender and age. Caregivers with income higher than 10 MW, living in houses with more than 3 people, and with graduate schooling showed higher EC scores. Caregivers considered competent eaters by ecSI2.0^TM^BR scores differed only for educational level, which was more frequent among graduate participants. The total EC score was positively associated with total and mealtime structure (D1), how food is available to the child (D3), and the parent gives respect to the child’s autonomy in eating (D4) sDOR.2-6y^TM^ domains. There was a negative association with the what is available to the child (D2) sDOR.2-6y^TM^ domain. In general, the sDOR.2-6y-BR had a positive association with the ecSI2.0^TM^BR in all domains and total, with a low but significant correlation. This study enables the investigation of the division of responsibility in feeding and EC of a sample of caregivers of children in Brazil. This is the first study to apply the translated and validated version of the sDOR.2-6y-BR and showed good results, where competent eaters’ caregivers adhere more to the principles of sDOR.

## 1. Introduction

The feeding practices built in childhood impact children’s growth and development and are likely to impact eating behavior throughout life [1]. Infant feeding is a process that requires skills and commitment from caregivers and children. To summarize this process, children demonstrate feeding-related signals and it is up to caregivers to interpret them. By relying on this to manage the child’s feeding, the caregiver reinforces the construction of the child’s autonomy to respond to their hunger and satiety signals, respecting their innate ability to self-regulate food intake [2,3].

**Figure 1:**
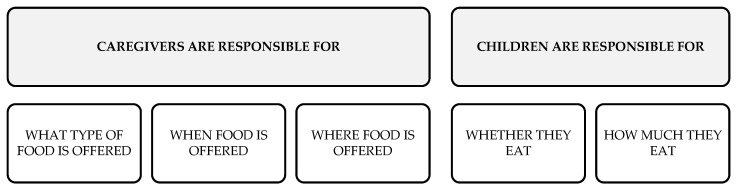
Graphical representation of the Division of Responsibility in Feeding [2].

The Division of Responsibility in Feeding (sDOR) was developed to help caregivers support children in developing a good relationship with themselves and with food, in the direction of building a healthy eating experience [3,4,5]. The sDOR is a model that is based on the division of tasks among caregivers and children (Figure 1) and states that caregivers provide structure and support without limiting the children’s autonomy to eat and power of choice and that caregivers’ eating habits serve as an example for the children [3,4,5,6,7]. The sDOR domains are: mealtime structure (D1), what is available to the child (D2), how food is available to the child (D3), the parent gives respect to the child’s autonomy in eating (D4), and who controls what, when, and how much is eaten (D5) [7,8].

The theoretical basis of sDOR presents data suggesting that children who receive appropriate support to develop autonomy in eating have more diverse food choices and balanced caloric intake. As a result, they grow and develop appropriately [7].

**Figure 2:**
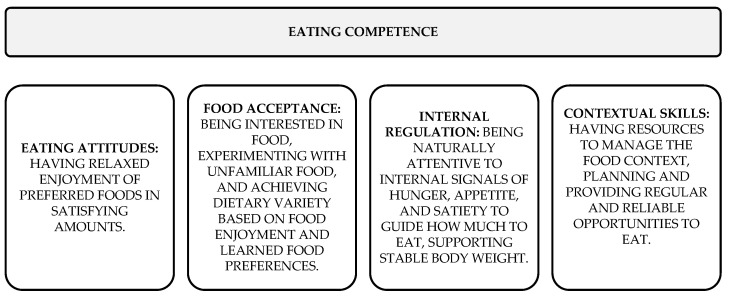
Graphical representation of the Eating Competence model components [9,10,11].

The Eating Competence model (EC) considers the biological, social, and psychological aspects of eating, and it gives autonomy to the individual to decide on what or how much to eat [9], precisely in the same way that sDOR postulates [3,4,5]. EC is divided into four components (as detailed in Figure 2): attitudes about eating and about food; food acceptance skills; internal regulation skills; and skills and resources for managing the food context and orchestrating family meals [9]. By following the principles of the sDOR, it is expected that the child will become a competent eater. Furthermore, parents classified with higher EC scores tend to follow more of the principles described in sDOR [7].

How caregivers feed children can determine important characteristics for developing and maintaining children’s health [12]. It is important to pay attention to the principles of EC and sDOR because how caregivers handle feeding strongly influences pre-school children’s eating behavior development and the ability to learn to enjoy nutritious and varied foods. Evidence shows that eating behaviors determined in early childhood persist into adulthood [4,13].

It is valid to expose that mothers considered competent eaters could be guided by their children’s hunger and satiety signals and consider their children eating well [14]. This study hypothesizes that parents or caregivers with greater adherence to the Division of Responsibility in Feeding have higher scores of Eating Competence. The other hypothesis is that female caregivers, and those with higher income and schooling, have higher adherence scores of Division of Responsibility in Feeding and higher scores of Eating Competence. 

This study aimed to assess caregiver adherence to sDOR and its EC. This study enables the investigation of the division of responsibility in feeding and eating competence of a sample of children’s caregivers in Brazil. Our study did not aim to evaluate eating or feeding disorders, but the biological, social, and psychological aspects of parents/child caregivers’ eating evaluated by the EC scores and the division of responsibility in child feeding to help caregivers support children in developing a good relationship with food and building a healthy eating experience. The data obtained have the potential to guide health professionals in the search for new strategies in the care of infant feeding and can assist in constructing new public policies related to the feeding of pre-school children.

## 2. Materials and Methods

This study is an exploratory, quantitative, cross-sectional with non-probability sampling, approved on 14 June 2022 by the Research Ethics Committee of the Faculty of Health Sciences of the University of Brasília, Federal District, Brazil (CAAE 56301222.1.0000.0030).

The research had national coverage in all regions of Brazil. The sample was composed of Brazilian citizens, mothers, fathers, or caregivers of Brazilian children aged 24 to 72 months. The exclusion criteria were fathers, mothers, or caregivers who were students in nutrition courses or graduates in nutrition. 

For data collection, we used a non-probability convenience sampling method of the virtual environment recruitment “snowball” type [15]. This method was selected considering the COVID-19 pandemic during the data collection period, limiting access to the face-to-face survey method. Studies have shown that “snowball” sampling via social media is an effective and efficient way to recruit study participants and allows for a larger sample size and shorter completion time, as well as a reduction in application costs [15,16]. Moreover, the choice of this method considered the one previously used in the original study that applied sDOR.2-6y^TM^ [7]. The participants’ participation in the study was completely voluntary.

The capture of participants was also carried out actively through different strategies. Coordinators and directors of schools, both public and private, were requested to help spread the survey to parents and caregivers of students. Dissemination was conducted on social networks and through the personal contacts of the research group members. People receiving the research link were encouraged to spread it to acquaintances who may fit the target audience. 

Data on sDOR and EC were collected using the sDOR.2-6y^TM^ Portuguese—Brazil and ecSI2.0^TM^BR, which measure sDOR adherence and can predict nutrition risk in children [7] and EC scores [10,11,17,18]. Both are the only validated instruments capable of assessing, respectively, sDOR and EC, and have already been translated into Brazilian Portuguese and validated in the Brazilian population [6,7,10,11,17,18]. All survey responses were scored according to the guidelines published in the original studies [6,7,8,17,19], and participant characteristics were reported using descriptive statistics.

For data collection, the survey components were entered into Google Forms©. On the initial page, there was the informed consent form, which provided the title of the research and its objectives, the option of the participant to refuse to participate without any prejudice, and the guarantee of confidentiality of the collected data. After that, the participant was asked a particular question about being a nutrition student or nutritionist, which, if answered affirmatively, automatically concluded the participation in the study. Sociodemographic questions were asked to those who went on to the following steps of the survey. After that, the respondents moved on to the items of the sDOR.2-6y-BR and then to the items of the—ecSI2.0^TM^BR. It is worth mentioning that the NEEDS center, the owner of the copyrights, previously authorized the use of the two questionnaires. The items of both questionnaires were arranged in the order indicated by the authors, as well as their response options.

For the statistical analysis, the floor and ceiling effects verified the questionnaire’s responsiveness. Floor effect is observed when sDOR.2-6y-BR (and its five components) produces a score equal to zero, and the ceiling effect occurs when the instrument (and its five components) reaches maximum values.

The scores of the sDOR.2-6y-BR (and its components) were described in terms of means, standard deviations (SD), medians, and interquartile range. Student’s *t*-test and analysis of variance (ANOVA) followed by Tukey’s post hoc tests were used to compare the scores of sDOR.2-6y-BR and ecSI2.0^TM^BR with interest variables. The Kolmogorov–Smirnov test verified the normality assumption. The results of the Satter Division of Responsibility in Feeding (sDOR ≥ 24) and categorized Eating Competence (ecSI ≥ 32) were described in terms of frequencies and percentages, and Pearson chi-squared tests verified its association with the variables of interest. The association between sDOR.2-6y-BR scores with ecSI2.0^TM^BR scores were verified by Pearson’s correlation coefficient. All tests were performed considering bilateral hypotheses and a 5% significance level. The analyses were performed using IBM SPSS (IBM SPSS Statistics for Windows, IBM Corp, Armonk, NY, USA) version 22. 

## 3. Results

### 3.1. Sociodemographic Data

The questionnaire with sociodemographic variables, the sDOR.2-6y^TM^ Portuguese (Brazil), and the ecSI2.0^TM^BR were available online from July 2022 to January 2023, using the Google Forms© platform. Of the 627 individuals who started answering the online survey, 78 did not provide all the necessary sociodemographic data for their participation (and did not reach the steps containing the sDOR.2-6y^TM^ Portuguese—Brazil and the ecSI2.0^TM^BR), and the final sample consisted of 549 participants who answered all the questions that made up the survey. Most of the caregivers who participated in the study were women (*n* = 478) with an average age of 37.8 years (standard deviation 5.1), a high school level (70.31%), and a high monthly income (more than 15 MW) (31.69%). The children for whom the participants were responsible were mostly girls (53.19%), with an average age of 3.6 years (standard deviation 1.3) (Supplementary file, Table S1).

### 3.2. sDOR.2-6y^TM^ Portuguese—Brazil

Table 1 shows sDOR.2-6y^TM^ Portuguese—Brazil scores and responsiveness of the questionnaire (*n* = 549, Brazil). Although domain D4 presents a ceiling effect >20%, the entire instrument presents good responsiveness (floor and ceiling effects = 0%). The internal consistency evaluation data were analyzed through the instrument’s Cronbach’s alpha coefficient (Cronbach’s alpha = 0.268). 

Concerning the sDOR.2-6y-BR scores, there was no statistical difference between the categories of caregiver’s gender, caregiver’s age, caregiver’s marital status (except for D5, where *p* = 0.049), caregiver’s level of education, number of people living in the household, nor by gender or age of the child (Table 2). Concerning the category of caregiver’s professional occupation, there was a statistical difference only for D2, where those with an occupation showed higher sDOR adherence scores than those without an occupation (Table 2). Regarding income, there was a statistical difference only for D2, and caregivers with income up to three minimum wages had lower sDOR adherence scores than those with income above six minimum wages (Table 2). 

Caregivers who reported (*n* = 100) that their children had some medical diagnosis (e.g., food allergy, autism, Down syndrome, etc.) had lower sDOR adherence scores than caregivers who indicated their children had no medical diagnosis (*p* = 0.031) (Table 2). The competent eaters’ caregivers presented higher sDOR adherence in domains 1, 3, 5, and total. 

### 3.3. ecSI2.0^TM^BR

Concerning the ecSI2.0^TM^BR scores, there was no statistical difference between the categories of caregiver’s gender, age, occupancy, and child’s gender and age. The categories of income (*p* = 0.007), number of people living in the household (*p* = 0.016), and caregiver’s schooling level (*p* = 0.011) showed a statistical difference for the food acceptance domain. Caregivers with income higher than 10 MW, living in houses with more than 3 people, and with graduate schooling education levels showed higher EC scores (Table 3). Caregivers with high school level education showed higher EC scores. The category of caregiver’s marital status showed statistical differences in the food acceptance domain (*p* = 0.009), contextual skills domain (*p* = 0.009), and the total score (*p* = 0.014). Caregivers who declared to have partners showed higher EC scores (Table 3). Caregivers considered competent eaters differed only for educational level, which was more frequent among graduate participants.

### 3.4. Associations between the sDOR.2-6y-BR and the ecSI2.0^TM^BR

Table 4 presents the associations between the sDOR.2-6y-BR and the ecSI2.0^TM^BR. The total EC score was positively associated with total and D1, D3, and D4 sDOR.2-6y^TM^ domains and presented a negative association with the D2 sDOR.2-6y^TM^ domain. The D2 sDOR.2-6y^TM^ domain had a negative association with the EC contextual skills domain and the total EC. The D4 sDOR.2-6y^TM^ domain was associated with the eating attitude, food acceptance, and total EC domains. The D5 sDOR.2-6y^TM^ domain was positively associated with food acceptance, contextual skills, and total EC score. In general, the sDOR.2-6y-BR had a positive association with the ecSI2.0^TM^BR in all domains and total EC, with a significant correlation.

## 4. Discussion

### 4.1. Sociodemographic Data

This study is the first to evaluate the division of responsibility in infant feeding and EC among Brazilian caregivers. Our findings show that most participants were female (88.7%), aged 37.8 ± 5.1 y/o. The reason for this result is probably that women tend to be more concerned with issues related to their health and children’s health for whom they are responsible [20,21]. A recent study conducted in Brazil with child caregivers, which investigated food neophobia, also obtained a high number (86%) of women among the participants [20]. It is also consistent with the original validation study of the sDOR.2-6y^TM^ in which most of the participants were female (94%) but with a lower mean age (32.2 ± 7.8 y/o) [7]. A recent study assessing the EC of caregivers in the United States had 94.8% of its audience composed of females [22]. In our study, children for whom the participants were responsible were nearly half girls (53.19%); this was similar to data from 2018 from the Brazilian Institute of Geography and Statistics, which reported similar distribution with almost half (49.1%) of children up to 12 years old in Brazil being girls [23].

The high education and income found in this study’s participants can be explained, in part, by the feature that the study was conducted entirely online. More developed regions tend to have more developed communication structures, with better access to the Internet, and Internet users tend to have a higher level of education and a higher monthly income [24,25,26]. People with a higher level of education are more likely to use online resources and to use them to search for health information [26]. Higher-income seems to be one of the main factors determining responses to health surveys [27]. The average per capita monthly income in Brazil in 2022, according to the Brazilian Institute of Geography and Statistics, was BRL 1625.00 (about USD 312.75), and more than a third of our study population had a monthly income of at least 11 times higher than that, which highlights the high-income levels of our participants [28]. Gender, educational, and income profiles show that our results cannot be extrapolated to all Brazilian caregivers, and further studies are needed to access a large and representative sample. However, it is the beginning of studies with the Brazilian population on the division of responsibilities in infant feeding, providing important data for future studies and helping health professionals guide child caregivers.

### 4.2. sDOR.2-6y^TM^ Portuguese—Brazil

The instrument as a whole presents good responsiveness (floor and ceiling effects = 0%), indicating it is sensitive to detect differences in the division of responsibility between caregivers located at the extremes (e.g., with better or worse scores) [29]. Although the internal consistency analysis of this study revealed a low Cronbach’s alpha value (0.268) of the sDOR.2-6y^TM^ Portuguese—Brazil, this value is close to those found in the original validation study of the sDOR.2-6y^TM^ (Cronbach’s alpha = 0.32) [7]. Because of the nature of the sDOR.2-6y^TM^ (twelve items divided into five domains), a low Cronbach’s alpha value is expected, as its value is directly affected by the instrument’s number of items and domains [30,31,32]. It is important to highlight that despite the low value, the sDOR.2-6y^TM^ has great importance in research in this area because it is the only valid instrument to measure adherence to sDOR.

The D5 of the sDOR.2-6y^TM^ that deals with “child autonomy with eating”, specifically about “who controls what, when, and how much is eaten”, showed a statistical difference for marital status, where caregivers who reported having partners had higher sDOR adherence scores. A study performed in the United States with 1839 parents of 2–5 y/o children who lived with them at least 50% of the time showed that there were less structured meals and they were less engaged with the child when they did not have partner support [33]. However, those who knew they would have partner support prepared more elaborate meals [33]. A systematic review on infant feeding highlighted that single-parent families might have more difficulties in cultivating healthy eating practices [34]. Another North American study, with 160 parents of 4 y/o children, reinforces that having a partnership to manage the child’s feeding can be linked with less pressure for the child to eat, with a greater organization of feeding and the promotion of the child’s autonomy [35].

Our study found that caregivers with professional occupations had higher sDOR scores than those without occupations. These data were possibly found because working parents tend to have less time to share mealtime with their families and less time for their children [34]. They tend to give greater autonomy and support to children to carry out daily activities, including eating [36]. It is important to emphasize that the child’s autonomy in eating must occur when the child’s choice is made up of healthy foods and not with what is easier, more practical, and often unhealthy. A review showed that working parents have more difficulty implementing healthy eating habits for their children due to their lack of time [34]. Another review on family income and its impact on children’s outcomes brought up the important reflection that, although working parents may have a better income, this may imply less time at home and less involvement with the child, showing the importance of the availability of healthy food at home [37].

Caregivers who did not pressure their children to eat had higher scores in the EC eating attitude and food acceptance domains. It addresses confidence in one’s ability to eat enough and enjoy eating, openness to try new foods, and to consume a wide variety of foods. A review of caregiver influences on eating behaviors in young children showed that when caregivers pressure a child to eat, the act is usually associated with good intentions. However, the habit of pressuring a child to eat also disrupts the child’s ability to self-regulate food intake. These data show that the caregivers in our study are acting to protect the child’s health since they favor the maintenance of self-management regulation by not pressuring the child to eat [38].

The “Feeding Guidelines for Infants and Young Toddlers” highlights that pressuring children to eat foods considered healthy, such as fruits and vegetables, can lead to an aversion to these foods and increased consumption of high-calorie sweet foods and snacks in pre-school children [39]. Adherence to sDOR was associated with less pressure to eat [7].

Caregivers who reported that their children had some medical diagnosis had lower sDOR adherence. This probably occurs due to the difficulty of trusting that the children’s choices will suit their needs without risking their health (e.g., contamination in case of food allergy, sensitivity, or intolerance; malnutrition in the case of highly selective behavior, such as autistic spectrum disorder). A cross-sectional study of 113 parents of 5–13 y/o ASD children showed that the children’s challenging eating behaviors (such as food selectivity, refusal to eat, and rigidity at mealtimes) were associated with high levels of parental stress [40], which makes it more difficult for parents to provide autonomy to the child in feeding. 

A review that addressed nutrition challenges in children and adolescents with Down syndrome found that parents put less pressure on their children to eat, were more concerned about their children’s weight, and imposed more restrictions on their children’s eating [41]. A review on feeding difficulties in children with non-IgE-mediated food allergy gastrointestinal disorders showed that caregivers of allergic children have higher anxiety levels and worse quality of life, which highlights that the anxiety of these caregivers can influence eating difficulties in children. It was presented that these parents are also afraid to feed their children [42], which can probably impact the ability to give autonomy to the child. The review also points out that adherence to sDOR is a strategy to make it easier for allergic children to recognize and trust safe foods [42]. A cross-sectional case-control study with 133 participants that investigated parenting promoting autonomy and independent problem-solving in children between 3 and 6 years of age with food allergies showed that because parents must have greater control of the food of the allergic child to minimize health risks, this action may end up generating uninteresting consequences, such as a difficulty for the child in developing autonomy in several areas of life [43]. It makes us reflect on the importance of educating these parents to be more secure and to be able to provide autonomy for their children in feeding. A cross-sectional study that applied semi-structured interviews to 15 parents of children with food hypersensitivity showed that caregivers used different strategies to promote their children’s autonomy. Among them, there is teaching children to explain effectively about their food restrictions from an early age because, at certain times, children would be in the absence of their caregivers and would need mechanisms to keep themselves safe concerning their food hypersensitivity. Knowing that the children were capable of this reassured the caregivers [44].

A study showed that children with specific medical diagnoses, such as dysphagia, should be supported by programs that promote safe mealtimes and a positive eating environment to avoid risks associated with their medical condition [45], corroborating with the sDOR principles. Despite not being explored in our study, the sDOR model might have applicability to health professionals in guiding caregivers in childhood feeding problems. However, further studies should be conducted in this field.

### 4.3. ecSI2.0^TM^BR

Similar to our study, a study conducted in Brazil in 2020, with 1810 adults, that investigated EC associated with health outcomes revealed that EC increased with income and education level, both in total and for food acceptance [46]. A review on EC highlighted that food acceptance is associated with social and economic factors and is a determinant of varied food intake [47]. In addition, a cross-sectional study conducted in 2014 in Japan with 3137 adult individuals aged 30–59 years showed that higher levels of income and education were associated with higher vegetable consumption, greater use of nutritional information, and meal commensality [48]. This can probably be linked to higher EC. The Brazilian study above cited also found higher fruit consumption among individuals with higher EC [46], corroborating our findings that higher income and education level are linked with higher EC.

A Taiwanese study performed in 2017 investigated the association of sociodemographic factors with EC in 564 elderly people. It showed that individuals who reported having partners showed significantly higher EC scores, and higher scores for food acceptance, just as in our study (although with a different target population age range) [49].

### 4.4. Associations between the sDOR.2-6y^TM^ Portuguese—Brazil and the ecSI2.0^TM^BR

The caregivers with higher eating competence scores were the ones who showed more skills in organizing their family’s meals, in giving autonomy to the child to make choices, and in following the previously established planning for their children’s meals. At the same time, they were the ones who pressured their children to eat. These findings are consistent with the original sDOR.2-6y^TM^ validation study, which showed that parents with more adherence to sDOR had higher EC scores, less habitual cognitive restraint in eating [7] (controlling food intake to control weight and body shape [50]), displayed a less authoritarian style of feeding their children, exercised fewer restrictions on their children’s eating, and placed less pressure on their children to eat [7].

Caregivers who demonstrate that they give in to the child’s food desires to plan the household meals had higher scores in the EC contextual skills domain, which deals precisely with food planning, including the abilities to plan, buy, and prepare their meals. A review on EC [47] showed that being able to cook facilitates the adoption of a healthier diet. Positive associations exist between individuals considered competent eaters and the habit of preparing meals at home and having pleasure in preparing them [47].

It was also shown that individuals with cooking habits presented a better food quality, with higher consumption of fruits, vegetables, and whole grains, for example. In addition, parents with cooking skills are more likely to offer their children a diet with less industrialized foods and have an important role in influencing their children’s consumption of fruits and vegetables [47].

Caregivers who scored higher in D5 also had the highest scores in the EC food acceptance domain, which deals with openness to try new foods, eating variety as well as eating foods they do not like but know are good for their health. Moreover, they had the highest scores in the contextual skills domain, which deals with abilities to organize their meals. These findings are consistent with a review on caregiver influences on eating behaviors in young children, which suggests that caregivers focus less on how much or what a child eats, but rather focus their energy on providing structure for varied eating, with diverse exposure to healthy foods, in an environment that fosters better food choices, and eating together with children to encourage them [38].

Our study has limitations that deserve to be listed, and caution should be exercised when interpreting and extrapolating the data presented here. Some biases due to the nature of the online study with a self-administered questionnaire are evident, such as a very homogeneous population (the result of recruitment with a non-probabilistic convenience sample) composed mainly of females, people with high financial conditions, and level of education.

## 5. Conclusions

This is the first Brazilian study to apply the translated and validated version of the sDOR.2-6y^TM^ Portuguese—Brazil, the only tool available to exclusively assess the division of responsibility in feeding. This study showed good results, similar to those found in other countries where competent eaters’ caregivers adhere more to the principles of sDOR. Our hypothesis that caregivers with greater adherence to the division of responsibility in feeding have higher eating competence scores was confirmed. Future studies with diverse populations are needed to examine the possible findings and minimize the limitations of the present study. We reinforce the importance of this study, which pioneered the development of data on adherence to the division of responsibility for feeding in the Brazilian population, and remind that these data can be important for health and education professionals who deal with pre-school age children and for authorities working in the development of public policies focused on the protection and promotion of children’s health and dissemination of knowledge about nutrition education.

## Figures and Tables

**Table 1:** The sDOR.2-6y^TM^ Portuguese—Brazil scores and responsiveness of the questionnaire (*n* = 549, Brazil).

Domains	Mean (SD)	Median (Q1–Q3)	Range	Floor Effect (%)	Ceiling Effect (%)
D1—Mealtime structure	4.49 (1.16)	5 (4–5)	0–6	0.2%	18.4%
D2—What is available to the child	3.58 (1.54)	4 (3–5)	0–6	5.1%	7.8%
D3—How food is available to the child	5.81 (1.41)	6 (5–7)	0–9	0.4%	2.4%
D4—Parent gives respect to the child’s autonomy in eating	4.18 (1.54)	5 (3–5)	0–6	2.0%	23.1%
D5—Who controls what, when, and how much is eaten	5.35 (1.57)	5 (4–6)	1–9	0%	1.6%
Total	23.41 (3.63)	24 (21–26)	10–33	0%	0%

**Table 2:** Sub-scores and categories of the sDOR.2-6y-BR ^1^ scales segregated by sociodemographic and economic characteristics (*n* = 549—Brazil).

	D1	D2	D3	D4	D5	Total	sDOR.2-6y-BR ^1^ ≥ 24 ****
	Mean (SD)	Mean (SD)	Mean (SD)	Mean (SD)	Mean (SD)	Mean (SD)	Freq (%)
Caregiver’s gender *							
Female (*n* = 487)	4.48 (1.16) ^A^	3.59 (1.54) ^A^	5.84 (1.40) ^A^	4.20 (1.57) ^A^	5.38 (1.57) ^A^	23.49 (3.64) ^A^	255 (52.4%) ^A^
Male (*n* = 62)	4.58 (1.11) ^A^	3.50 (1.50) ^A^	5.60 (1.44) ^A^	4.05 (1.31) ^A^	5.08 (1.49) ^A^	22.81 (3.55) ^A^	27 (43.5%) ^A^
*p*	0.504	0.547	0.204	0.407	0.156	0.165	0.191 ***
Caregiver’s age *							
Up to 39 years (*n* = 348)	4.50 (1.08) ^A^	3.56 (1.58) ^A^	5.80 (1.40) ^A^	4.20 (1.55) ^A^	5.29 (1.48) ^A^	23.34 (3.55) ^A^	174 (50.0%) ^A^
40 years or more (*n* = 201)	4.47 (1.27) ^A^	3.63 (1.45) ^A^	5.83 (1.41) ^A^	4.16 (1.54) ^A^	5.45 (1.71) ^A^	23.53 (3.77) ^A^	108 (53.7%) ^A^
*p*	0.752	0.610	0.797	0.791	0.266	0.549	0.399 ***
Caregiver’s marital status *							
With partner (*n* = 485)	4.48 (1.18) ^A^	3.59 (1.51) ^A^	5.82 (1.40) ^A^	4.15 (1.55) ^A^	5.39 (1.57) ^B^	23.44 (3.68) ^A^	
Without partner (*n* = 64)	4.58 (0.99) ^A^	3.53 (1.70) ^A^	5.70 (1.45) ^A^	4.39 (1.47) ^A^	4.98 (1.50) ^A^	23.19 (3.26) ^A^	35 (54.7%)
*p*	0.508	0.775	0.516	0.250	0.049	0.603	0.572 ***
Caregiver’s schooling level **							
High School (*n* = 28)	4.61 (0.96) ^A^	2.93 (1.82) ^A^	5.89 (1.31) ^A^	4.07 (1.59) ^A^	4.96 (1.20) ^A^	22.46 (3.26) ^A^	12 (42.9%) ^A^
Undergraduate (*n* = 135)	4.53 (1.15) ^A^	3.61 (1.48) ^A^	5.73 (1.34) ^A^	4.22 (1.45) ^A^	5.28 (1.56) ^A^	23.37 (3.55) ^A^	70 (51.9%) ^A^
Graduate (*n* = 386)	4.46 (1.17) ^A^	3.62 (1.52) ^A^	5.83 (1.44) ^A^	4.18 (1.57) ^A^	5.40 (1.59) ^A^	23.49 (3.68) ^A^	200 (51.8%) ^A^
*p*	0.714	0.068	0.707	0.887	0.318	0.349	0.652 ***
Caregiver’s occupancy *							
With occupancy (*n* = 502)	4.46 (1.16) ^A^	3.64 (1.51) ^B^	5.79 (1.41) ^A^	4.20 (1.54) ^A^	5.33 (1.55) ^A^	23.42 (3.64) ^A^	258 (51.4%) ^A^
Without occupancy (*n* = 47)	4.79 (1.08) ^A^	3.02 (1.71) ^A^	6.02 (1.34) ^A^	3.96 (1.52) ^A^	5.53 (1.73) ^A^	23.32 (3.57) ^A^	24 (51.1%) ^A^
*p*	0.063	0.009	0.283	0.297	0.395	0.858	0.965 ***
Income ^+,^***							
Up to 3 MW (*n* = 47)	4.60 (1.08) ^A^	2.91 (1.50) ^A^	5.91 (1.36) ^A^	4.02 (1.65) ^A^	5.09 (1.56) ^A^	22.53 (2.96) ^A^	19 (40.4%) ^A^
4 to 5 MW (*n* = 46)	4.50 (1.17) ^A^	3.50 (1.86) ^AB^	5.72 (1.33) ^A^	4.37 (1.50) ^A^	5.26 (1.36) ^A^	23.35 (3.92) ^A^	23 (50.0%) ^A^
6 to 9 MW (*n* = 89)	4.49 (1.10) ^A^	3.74 (1.56) ^B^	5.82 (1.38) ^A^	4.31 (1.47) ^A^	5.04 (1.54) ^A^	23.42 (3.28) ^A^	46 (51.7%) ^A^
10 to 15 MW (*n* = 146)	4.63 (1.16) ^A^	3.72 (1.57) ^B^	5.82 (1.47) ^A^	4.21 (1.52) ^A^	5.42 (1.49) ^A^	23.80 (3.60) ^A^	84 (57.5%) ^A^
More than 15 MW (*n* = 174)	4.35 (1.11) ^A^	3.64 (1.32) ^B^	5.78 (1.44) ^A^	4.06 (1.56) ^A^	5.54 (1.59) ^A^	23.37 (3.73) ^A^	85 (48.9%) ^A^
*p*	0.254	0.020	0.968	0.554	0.089	0.328	0.293 ***
Number of people living in the household **							
2 (*n* = 35)	4.71 (0.93) ^A^	3.54 (1.65) ^A^	5.34 (1.39) ^A^	4.37 (1.48) ^A^	4.91 (1.54) ^A^	22.89 (3.59) ^A^	18 (51.4%) ^A^
3 (*n* = 227)	4.44 (1.18) ^A^	3.56 (1.44) ^A^	5.95 (1.48) ^A^	4.27 (1.60) ^A^	5.26 (1.51) ^A^	23.49 (3.69) ^A^	120 (52.9%) ^A^
4 (*n* = 218)	4.53 (1.15) ^A^	3.56 (1.60) ^A^	5.75 (1.38) ^A^	4.07 (1.51) ^A^	5.44 (1.60) ^A^	23.35 (3.55) ^A^	110 (50.5%) ^A^
5 or more (*n* = 69)	4.39 (1.19) ^A^	3.72 (1.61) ^A^	5.78 (1.17) ^A^	4.14 (1.52) ^A^	5.55 (1.61) ^A^	23.59 (3.76) ^A^	34 (49.3%) ^A^
*p*	0.495	0.879	0.092	0.470	0.149	0.786	0.941 ***
Child’s gender *							
Female (*n* = 292)	4.51 (1.11) ^A^	3.52 (1.61) ^A^	5.87 (1.42) ^A^	4.11 (1.58) ^A^	5.37 (1.57) ^A^	23.37 (3.63) ^A^	155 (53.1%) ^A^
Male (*n* = 257)	4.46 (1.21) ^A^	3.66 (1.45) ^A^	5.75 (1.39) ^A^	4.27 (1.49) ^A^	5.32 (1.57) ^A^	23.45 (3.65) ^A^	127 (49.4%) ^A^
*p*	0.581	0.285	0.321	0.219	0.705	0.802	0.391 ***
Child’s age **							
2 years (*n* = 149)	4.47 (1.21) ^A^	3.47 (1.53) ^A^	5.93 (1.39) ^A^	4.25 (1.60) ^A^	5.56 (1.54) ^A^	23.67 (3.68) ^A^	80 (53.7%) ^A^
3 years (*n* = 123)	4.59 (1.14) ^A^	3.46 (1.52) ^A^	5.78 (1.28) ^A^	4.16 (1.40) ^A^	5.34 (1.49) ^A^	23.33 (3.34) ^A^	62 (50.4%) ^A^
4 years (*n* = 128)	4.49 (1.10) ^A^	3.66 (1.50) ^A^	5.59 (1.44) ^A^	4.18 (1.52) ^A^	5.18 (1.64) ^A^	23.10 (3.95) ^A^	63 (49.2%) ^A^
5 years (*n* = 81)	4.40 (1.06) ^A^	3.70 (1.68) ^A^	5.85 (1.60) ^A^	4.04 (1.65) ^A^	5.21 (1.52) ^A^	23.20 (3.70) ^A^	38 (46.9%) ^A^
6 years (*n* = 68)	4.44 (1.29) ^A^	3.76 (1.48) ^A^	5.99 (1.31) ^A^	4.25 (1.59) ^A^	5.37 (1.63) ^A^	23.81 (3.38) ^A^	39 (57.4%) ^A^
*p*	0.792	0.483	0.244	0.888	0.331	0.586	0.695 ***
Child’s medical diagnosis *							
Yes (*n* = 100)	4.58 (1.01) ^A^	3.34 (1.70) ^A^	5.72 (1.42) ^A^	3.91 (1.71) ^A^	5.15 (1.44) ^A^	22.70 (3.78) ^A^	43 (43.0%) ^A^
No (*n* = 449)	4.47 (1.19) ^A^	3.64 (1.49) ^A^	5.83 (1.40) ^A^	4.24 (1.50) ^A^	5.39 (1.59) ^A^	23.57 (3.58) ^B^	239 (53.2%) ^A^
*p*	0.380	0.080	0.477	0.075	0.166	0.031	0.064 ***
ecSI2.0^TM^BR *							
≥32 ***** (*n* = 321)	4.74 (1.04) ^B^	3.49 (1.55) ^A^	6.13 (1.32) ^B^	4.25 (1.54) ^A^	5.47 (1.62) ^B^	24.07 (3.54) ^B^	191 (59.5%) ^B^
<32 (*n* = 228)	4.14 (1.22) ^A^	3.72 (1.50) ^A^	5.36 (1.41) ^A^	4.08 (1.55) ^A^	5.18 (1.47) ^A^	22.48 (3.57) ^A^	91 (39.9%) ^A^
*p*	<0.001	0.079	<0.001	0.206	0.029	<0.001	<0.001 ***

^1^ sDOR.2-6y^TM^ Portuguese—Brazil. * Student *t*-test. ** Anova with Tukey post hoc test. Groups with the same letters (A, B) do not differ significantly. *** Pearson chi-square test. **** A score of 24 or higher generally represents very good adherence to sDOR. ***** A score of 32 or higher indicates that the individual is considered a competent eater. ^+^ 1 MW = BRL 1212.00 (BRL: Brazilian Real is the official currency of Brazil and USD 1.00 = BRL 5.24, 28 February 2023). Groups with the same letters do not differ significantly. Note: The sum can be less than 549 due to the presence of missing values. D1—Mealtime structure. D2—What is available to the child. D3—How food is available to the child. D4—Parent gives respect to the child’s autonomy in eating. D5—Who controls what, when, and how much is eaten.

**Table 3:** Sub-scores and categories of the ecSI2.0^TM^BR scales segregated by sociodemographics and clinical characteristics (*n* = 549—Brazil).

	Eating Attitude	Food Acceptance	Internal Regulation	Contextual Skills	Total	ecSI2.0^TM^BR ≥32 ****
	Mean (SD)	Mean (SD)	Mean (SD)	Mean (SD)	Mean (SD)	Freq (%)
Caregiver’s gender *						
Female (*n* = 487)	12.49 (3.25) ^A^	5.64 (2.29) ^A^	4.07 (1.45) ^A^	10.33 (2.97) ^A^	32.54 (7.63) ^A^	286 (58.7%) ^A^
Male (*n* = 62)	13.29 (2.75) ^A^	5.45 (2.15) ^A^	4.32 (1.23) ^A^	10.32 (3.02) ^A^	33.39 (6.82) ^A^	35 (56.5%) ^A^
*p*	0.065	0.538	0.190	0.984	0.403	0.732 ***
Caregiver’s age *						
Up to 39 years (*n* = 348)	12.51 (3.19) ^A^	5.59 (2.27) ^A^	4.05 (1.46) ^A^	10.16 (2.92) ^A^	32.30 (7.42) ^A^	193 (55.5%) ^A^
40 years or more (*n* = 201)	12.71 (3.23) ^A^	5.67 (2.29) ^A^	4.19 (1.37) ^A^	10.63 (3.04) ^A^	33.20 (7.73) ^A^	128 (63.7%) ^A^
*p*	0.482	0.683	0.259	0.070	0.177	0.060 ***
Caregiver’s marital status *						
With a partner (*n* = 485)	12.64 (3.17) ^A^	5.71 (2.27) ^B^	4.12 (1.39) ^A^	10.45 (2.96) ^B^	32.92 (7.48) ^B^	290 (59.8%) ^A^
Without a partner (*n* = 64)	12.20 (3.43) ^A^	4.92 (2.25) ^A^	3.92 (1.67) ^A^	9.42 (2.96) ^A^	30.47 (7.68) ^A^	31 (48.4%) ^A^
*p*	0.311	0.009	0.294	0.009	0.014	0.083 ***
Caregiver’s schooling level **						
High School (*n* = 28)	13.00 (2.89) ^A^	4.75 (2.59) ^A^	4.25 (1.55) ^A^	9.50 (2.94) ^A^	31.50 (7.04) ^A^	12 (42.9%) ^A^
Undergraduate (*n* = 135)	12.13 (3.36) ^A^	5.30 (2.23) ^AB^	3.97 (1.56) ^A^	10.10 (3.23) ^A^	31.51 (8.02) ^A^	71 (52.6%) ^AB^
Graduate (*n* = 386)	12.71 (3.16) ^A^	5.79 (2.25) ^B^	4.13 (1.37) ^A^	10.47 (2.88) ^A^	33.11 (7.37) ^A^	238 (61.7%) ^B^
*p*	0.152	0.011	0.447	0.149	0.076	0.042 ***
Caregiver’s occupancy *						
With occupancy (*n* = 502)	12.65 (3.21) ^A^	5.65 (2.29) ^A^	4.11 (1.42) ^A^	10.30 (2.98) ^A^	32.71 (7.51) ^A^	296 (59.0%) ^A^
Without occupancy (*n* = 47)	11.89 (3.09) ^A^	5.26 (2.15) ^A^	4.02 (1.57) ^A^	10.62 (2.95) ^A^	31.79 (7.93) ^A^	25 (53.2%) ^A^
*p*	0.122	0.252	0.699	0.489	0.422	0.443 ***
Income ^+,^**						
Up to 3 MW (*n* = 47)	12.70 (3.32) ^A^	4.89 (2.47) ^AB^	4.06 (1.57) ^A^	10.19 (3.40) ^AB^	31.85 (8.95) ^A^	23 (48.9%) ^A^
4 to 5 MW (*n* = 46)	12.22 (3.78) ^A^	4.74 (2.53) ^A^	3.83 (1.90) ^A^	8.93 (3.37) ^A^	29.72 (9.04) ^A^	21 (45.7%) ^A^
6 to 9 MW (*n* = 89)	12.92 (2.96) ^A^	5.70 (2.07) ^AB^	4.08 (1.51) ^A^	10.16 (2.90) ^AB^	32.85 (6.94) ^A^	54 (60.7%) ^A^
10 to 15 MW (*n* = 146)	12.50 (3.20) ^A^	5.79 (2.34) ^B^	4.10 (1.43) ^A^	10.47 (2.90) ^B^	32.87 (7.42) ^A^	85 (58.2%) ^A^
More than 15 MW (*n* = 174)	12.61 (3.16) ^A^	5.86 (2.25) ^B^	4.14 (1.21) ^A^	10.63 (2.77) ^B^	33.25 (6.97) ^A^	109 (62.6%) ^A^
*p*	0.785	0.007	0.769	0.013	0.067	0.180 ***
Number of people living in the household **						
2 (*n* = 35)	12.40 (3.57) ^A^	4.63 (2.14) ^A^	4.09 (1.56) ^A^	9.69 (2.54) ^A^	30.80 (7.07) ^A^	17 (48.6%) ^A^
3 (*n* = 227)	12.79 (3.18) ^A^	5.87 (2.29) ^B^	4.17 (1.40) ^A^	10.63 (2.83) ^A^	33.46 (7.20) ^A^	142 (62.6%) ^A^
4 (*n* = 218)	12.42 (3.23) ^A^	5.61 (2.22) ^B^	4.04 (1.46) ^A^	10.20 (3.02) ^A^	32.26 (7.76) ^A^	125 (57.3%) ^A^
5 or more (*n* = 69)	12.52 (3.04) ^A^	5.35 (2.32) ^AB^	4.07 (1.36) ^A^	10.07 (3.42) ^A^	32.01 (8.01) ^A^	37 (53.6%) ^A^
*p*	0.655	0.016	0.811	0.174	0.123	0.289 ***
Child’s gender *						
Female (*n* = 292)	12.60 (3.35) ^A^	5.66 (2.26) ^A^	4.15 (1.44) ^A^	10.24 (2.97) ^A^	32.64 (7.63) ^A^	172 (58.9%) ^A^
Male (*n* = 257)	12.57 (3.03) ^A^	5.58 (2.29) ^A^	4.04 (1.42) ^A^	10.43 (2.98) ^A^	32.62 (7.45) ^A^	149 (58.0%) ^A^
*p*	0.931	0.675	0.361	0.450	0.969	0.826 ***
Child’s age **						
2 years (*n* = 149)	13.17 (3.03) ^A^	5.78 (2.23) ^A^	4.18 (1.48) ^A^	10.69 (2.80) ^A^	33.83 (6.89) ^A^	99 (66.4%) ^A^
3 years (*n* = 123)	12.21 (3.32) ^A^	5.78 (2.40) ^A^	4.15 (1.38) ^A^	10.44 (2.84) ^A^	32.59 (7.67) ^A^	70 (56.9%) ^A^
4 years (*n* = 128)	12.63 (3.13) ^A^	5.54 (2.25) ^A^	3.89 (1.37) ^A^	10.07 (3.40) ^A^	32.13 (8.17) ^A^	73 (57.0%) ^A^
5 years (*n* = 81)	12.21 (3.32) ^A^	5.49 (2.09) ^A^	4.06 (1.51) ^A^	9.81 (2.83) ^A^	31.58 (7.18) ^A^	42 (51.9%) ^A^
6 years (*n* = 68)	12.32 (3.24) ^A^	5.28 (2.42) ^A^	4.25 (1.41) ^A^	10.44 (2.81) ^A^	32.29 (7.74) ^A^	37 (54.4%) ^A^
*p*	0.080	0.515	0.378	0.209	0.198	0.195 ***
Child’s medical diagnosis *						
Yes (*n* = 100)	12.18 (3.11) ^A^	5.35 (2.36) ^A^	3.98 (1.43) ^A^	10.14 (3.11) ^A^	31.65 (7.42) ^A^	53 (53.0%) ^A^
No (*n* = 449)	12.67 (3.22) ^A^	5.68 (2.25) ^A^	4.12 (1.43) ^A^	10.37 (2.94) ^A^	32.85 (7.56) ^A^	268 (59.7%) ^A^
*p*	0.163	0.191	0.360	0.481	0.150	0.220 ***
sDOR.2-6y-BR ^1,^*						
≥24 ***** (*n* = 282)	12.99 (2.95) ^B^	6.06 (2.12) ^B^	4.20 (1.42) ^A^	10.90 (2.77) ^B^	34.16 (6.82) ^B^	191 (67.7%) ^B^
<24 (*n* = 267)	12.15 (3.41) ^A^	5.16 (2.34) ^A^	3.99 (1.43) ^A^	9.72 (3.07) ^A^	31.02 (7.94) ^A^	130 (48.7%) ^A^
*p*	0.002	<0.001	0.080	<0.001	<0.001	<0.001 ***

^1^ sDOR.2-6y^TM^ Portuguese—Brazil. * Student *t*-test. ** Anova with Tukey post hoc test. Groups with the same letters (A, B) do not differ significantly. *** Pearson chi-square test. **** A score of 32 or higher indicates that the individual is considered a competent eater. ***** A score of 24 or higher generally represents very good adherence to sDOR. ^+^ 1 MW = BRL 1212.00 (BRL: Brazilian Real is the official currency of Brazil and USD 1.00 = BRL 5.24, 28 February 2023). Groups with the same letters do not differ significantly. Note: The sum can be less than 549 due to the presence of missing values.

**Table 4:** Associations between sDOR.2-6y-BR ^1^ and ecSI2.0^TM^BR scores (and their domains) (*n* = 549—Brazil).

			ecSI2.0^TM^BR		
sDOR.2-6y-BR ^1^	Eating Attitude Pearson Correlation (*p*)	Food Acceptance Pearson Correlation (*p*)	Internal Regulation Pearson Correlation (*p*)	Contextual Skills Pearson Correlation (*p*)	Total Pearson Correlation (*p*)
D1	0.120 (0.005)	0.123 (0.004)	0.123 (0.04)	0.327 (<0.001)	0.240 (<0.001)
D2	−0.065 (0.128)	−0.011 (0.805)	−0.036 (0.398)	−0.121 (0.004)	−0.085 (0.045)
D3	0.231 (<0.001)	0.235 (<0.001)	0.163 (<0.001)	0.256 (<0.001)	0.301 (<0.001)
D4	0.156 (<0.001)	0.109 (0.010)	0.008 (0.859)	0.023 (0.589)	0.110 (0.010)
D5	−0.031 (0.469)	0.121 (0.004)	0.061 (0.156)	0.119 (0.005)	0.085 (0.056)
Total	0.156 (<0.001)	0.224 (<0.001)	0.116 (<0.001)	0.212 (<0.001)	0.238 (<0.001)

^1^ sDOR.2-6y^TM^ Portuguese—Brazil. D1—Mealtime structure. D2—What is available to the child. D3—How food is available to the child. D4—Parent gives respect to the child’s autonomy in eating. D5—Who controls what, when, and how much is eaten.

## Data Availability

Not applicable.

## Supplementary Materials

The following supporting information can be downloaded at: https://www.mdpi.com/article/10.3390/nu15092225/s1, Table S1: Sociodemographic characteristics of the individuals (*n* = 549—Brazil).
