# Supplementary material for: Division of Responsibility in Child Feeding and Eating Competence among Brazilian Caregivers
Source: Nutrients. 2023 May 8;15(9):2225. doi: 10.3390/nu15092225 (PMC10180630; doi:10.3390/nu15092225)
Supplement: Supplementary file 1 [file nutrients-15-02225-s001.zip › nutrients-2360515-supplementary.pdf]

Supplementary Files

Table S1. Sociodemographic characteristics of the individuals (n = 549 - Brazil).

		Sample (n = 549)
		Freq (%)
Caregiver's gender	Female	487 (87.07%)
	Male	62 (11.29%)
Caregiver's age	Up to 39 years	348 (63.39%)
	40 years or more	201 (36.61%)
Caregiver's schooling level	High School	28 (5.10%)
	Undergraduate	135 (24.59%)
	Graduate	386 (70.31%)
Income *	Up to 3 MW	47 (8.56%)
	4 to 5 MW	46 (8.38%)
	6 to 9 MW	89 (16.21%)
	10 to 15 MW	146 (26.59%)
	More than 15 MW	174 (31.69%)
Child's gender	Female	292 (53.19%)
	Male	257 (46.81%)
Child's age	2 years	149 (27.14%)
	3 years	123 (22.40%)
	4 years	128 (23.32%)
	5 years	81 (14.75%)
	6 years	68 (12.39%)

* 1 MW = BRL $ 1212.00 (BRL: Brazilian Real is the official currency of Brazil and 1.00 USD = 5.24 BRL – February 28 2023).
